# Experimental and theoretical studies of Schiff bases as corrosion inhibitors

**DOI:** 10.1186/s13065-018-0376-7

**Published:** 2018-02-05

**Authors:** Dalia M. Jamil, Ahmed K. Al-Okbi, Shaimaa B. Al-Baghdadi, Ahmed A. Al-Amiery, Abdulhadi Kadhim, Tayser Sumer Gaaz, Abdul Amir H. Kadhum, Abu Bakar Mohamad

**Affiliations:** 1 Chemistry Department, College of Science, University of Nahrain, Baghdad, Iraq; 2 University of Technology (UOT), Baghdad, 10001 Iraq; 3 Department of Chemical & Process Engineering, Universiti Kebangsaan Malaysia (UKM), 43000 Bangi, Selangor Malaysia

**Keywords:** Schiff bases, Corrosion inhibitors, SEM, NMR, DFT

## Abstract

**Background:**

Relatively inexpensive, stable Schiff bases, namely 3-((4-hydroxybenzylidene)amino)-2-methylquinazolin-4(3*H*)-one (BZ3) and 3-((4-(dimethylamino)benzylidene)amino)-2-methylquinazolin-4(3*H*)-one (BZ4), were employed as highly efficient inhibitors of mild steel corrosion by corrosive acid.

**Findings:**

The inhibition efficiencies were estimated based on weight loss method. Moreover, scanning electron microscopy was used to investigate the inhibition mechanism. The synthesized Schiff bases were characterized by Fourier transform infrared spectroscopy, nuclear magnetic resonance spectroscopy and micro-elemental analysis. The inhibition efficiency depends on three factors: the amount of nitrogen in the inhibitor, the inhibitor concentration and the inhibitor molecular weight.

**Conclusions:**

Inhibition efficiencies of 96 and 92% were achieved with BZ4 and BZ3, respectively, at the maximum tested concentration. Density functional theory calculations of BZ3 and BZ4 were performed to compare the effects of hydroxyl and *N*,*N*-dimethylamino substituents on the inhibition efficiency, providing insight for designing new molecular structures that exhibit enhanced inhibition efficiencies.

## Introduction

Anti-corrosion coatings are generally employed to inhibit the average of corrosion and increase longevity of the mild steel. A broad range of organic adsorption inhibitors presently applied in the corrosion domain are expensive [1, 2]. Electron pairs and negative ions are transferred from the inhibitors to the metal d orbitals, resulting in the formation of coordination complexes with specific geometries, such as square planar, tetrahedral or octahedral [3]. Thus, inhibitor molecules improve mild steel resistance to corrosive solutions by adsorbing on the metal surface [4–7] and forming a barrier that blocks the mild steel active sites [8–10]. Inhibitor adsorption on mild steel is affected by the nature of the mild steel, type of electrolyte and molecular structure of the inhibitor [11, 12]. Inhibitors molecules adsorbed on surface of mild steel, forming a barrier and consequently preventing reactions (cathodic or anodic) from processing at the surface of mild steel. These inhibitors could react with the iron atom at the mild steel surface to form in-organic complexes, blocking the surface of mild steel [13]. Quantum chemical investigations have extensively been employed for correlating the inhibitor molecular structures and the inhibition impacts [14]. To extend our previous work on designing novel inhibitor molecules [15–24], the Schiff bases 3-((4-hydroxybenzylidene)amino)-2-methylquinazolin-4(3*H*)-one (BZ3) and 3-((4-(dimethylamino)benzylidene)amino)-2-methylquinazolin-4(3*H*)-one (BZ4) were synthesized. Their molecular structures were determined by elemental analysis; carbon, hydrogen and nitrogen (mass fractions of CHN) analysis, Fourier transform infrared FTIR spectroscopy and nuclear magnetic resonance (NMR) spectroscopy. The abilities of these molecules to inhibit mild steel corrosion in an acidic solution were determined by the weight loss method and scanning electron microscopy (SEM). To elucidate the inhibition mechanism and the relationship between the structure and inhibition efficiency of the inhibitor, quantum chemical calculations of BZ3 and BZ4 were performed.

## Experimental

### Materials

All chemical compounds were purchased from Sigma-Aldrich/Malaysia. Fourier transform infrared (FTIR) spectra were recorded on a Shimadzu FTIR-8300 spectrometer. Elemental analyses were performed using a Carlo Erba 5500 elemental analysis; carbon, hydrogen and nitrogen (CHN). Nuclear magnetic resonance spectra were obtained using a Bruker Spectrospin instrument equipped with 300 MHz UltraShield magnets. DMSO-d6 and TMS were used as the solvent and internal standard, respectively.

### Synthesis of corrosion inhibitors

An ethanolic solution of 3-amino-2-methylquinazolin-4(3*H*)-one (0.005 mol), the appropriate carbonyl compound (0.005 mol) and a few drops of acetic acid were refluxed for 8 h. After cooling, the mixture was filtered, and the obtained solid was subsequently washed and recrystallized from hot ethanol. BZ3: yield 72%, mp 204–206 °C. FTIR: 3189 (br, aromatic O–H), 1704.3 (C=O), 1609.0 (C=N). ^1^H NMR: 2.37 (s, 3H, CH_3_), 6.84–7.01 (m, 1H, Ar–H), 5.32 (s, 1H, OH), 9.33 (d, 1H, H–C=N). Elemental analysis (CHN): C 69.11% (68.81%), H 4.91% (4.69%), N 14.82 (15.05). BZ4: yield 68%, mp 191–193 °C. FTIR: 3047.4 (aromatic C–H), 1699.6 (C=O), 1611.3 (C=N). ^1^H NMR: 2.410 (s, 3H, CH_3_), 7.01–7.32 (m, 1H, Ar–H), 8.99 (d, 1H, H–C=N). Elemental analysis (CHN): C 70.90% (70.57%), H 6.03% (5.92%), N 18.78 (18.29%).

### Corrosion tests

The mild steel specimens that were utilized as electrodes in this study were supplied by Metal Samples Company. The mild steel composition was 99.21% Fe, 0.21% C, 0.38% Si, 0.09% P, 0.05% S, 0.05% Mn and 0.01% Al. The mild steel effective area was 4.5 cm^2^, and the surface was cleaned according to ASTM G1-03 [25–27]. In a typical procedure, an mild steel sample was suspended (in duplicate) in 200 mL of a corrosive solution with or without an inhibitor (BZ3 and BZ4). The inhibitor concentrations studied were 0.001, 0.05, 0.10, 0.15, 0.2.0, 0.25 and 0.50 g/L. After a given amount of time (1, 2, 3, 4, 5, 10, 24, 48 and 72 h), the sample was washed, dried, and weighed. The inhibition efficiencies (IEs, %) were calculated using Eq. 1:

1 $${\text{IE}}\,\left( {\% } \right)\, = \,\left( {1 - \frac{{W_{2} }}{{W_{1} }}} \right)\, \times \,100$$

where W_1_ and W_2_ are the weight losses of the mild steel specimens in the absence and presence of an inhibitor, respectively.

### Calculation method

Ground-state geometry optimizations were performed without symmetry constraints using Gaussian 09, Revision A.02 [28]. The hybrid functional B3LYP was employed for all the geometry optimizations and highest occupied and lowest unoccupied molecular orbital energy calculations [29, 30].

## Results and discussion

### Synthesis

**Scheme 1:**
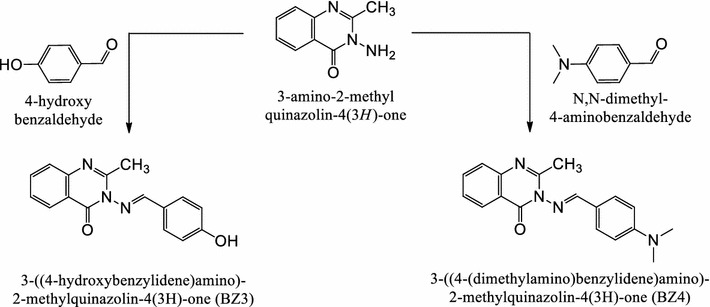
Inhibitors synthesis procedure

The Schiff bases BZ3 and BZ4 were readily synthesized in excellent yields by refluxing 3-amino-2-methylquinazolin-4(3*H*)-one with 4-hydroxybenzaldehyde and *N*,*N*-dimethyl-4-aminobenzaldehyde, respectively. The molecular weights of BZ3 and BZ4 were estimated to be 279 and 306, respectively, from the chemical formulas (C_16_H_13_N_3_O_2_ and C_18_H_18_N_4_O, respectively) and were confirmed by spectroscopic techniques. No hydrazide absorption bands were observed in the BZ3 and BZ4 FTIR spectra. The BZ3 ^1^H NMR (nuclear magnetic resonance) spectrum exhibited singlets at δ 5.32 ppm, due to the OH proton, and δ 2.37 ppm (3H), due to the methyl group. In the BZ4 ^1^H NMR spectrum, only one singlet was observed at δ 2.410 (3H) due to the methyl group. The Schiff bases were synthesized from 3-amino-2-methylquinazolin-4(3*H*)-one according to the procedure illustrated in Scheme 1.

### Weight loss results

In industry, the use of inhibitors is one of the major economical methods for efficiently safeguarding mild steel surfaces against corrosion [31]. Organic inhibitors are the predominant compounds used in the oil industry because they can act as a barrier for mild steel against corrosive media. Most of these inhibitors are heterocyclic molecules, such as pyridine, imidazoline and azoles [32–34], or polymers [35, 36].

### Concentration effect

**Fig. 1:**
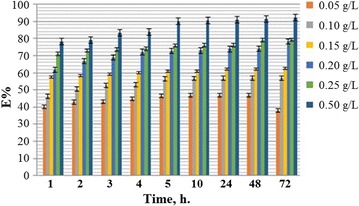
BZ3 inhibition efficiency for mild steel as a function of time at various inhibitor concentrations and 303 K

**Fig. 2:**
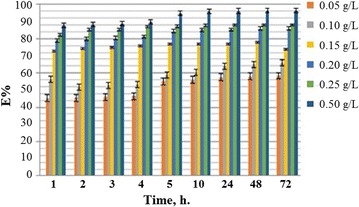
BZ4 inhibition efficiency for mild steel as a function of time at various inhibitor concentrations and 303 K

The weight loss method was used to calculate the inhibition efficiencies of, BZ3 and BZ4 at various concentrations (0.05, 0.1, 0.15, 0.2, 0.25 and 0.5 g/L) for (1, 2, 3, 4, 5, 10, 24, 48 and 72 h) and 303 K for mild steel in corrosive media. The BZ3 and BZ4 results, which are shown in Figs. 1 and 2, respectively, indicate that these inhibitors reduced mild steel corrosion in corrosive media. For all the inhibitors, the inhibition efficiency increased with increasing concentration, reaching a maximum at the highest tested concentration.

### Temperature effect

**Fig. 3:**
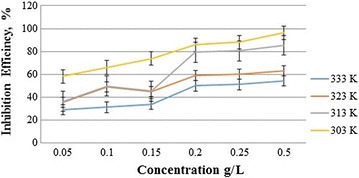
BZ4 inhibition efficiency as a function of the inhibitor concentration at various temperatures

To determine the effect of the temperature on the inhibition efficiency, corrosion experiments were performed in the absence or presence of BZ4 at various temperatures (303, 313, 323 and 333 K). The inhibition performance was enhanced by increasing the BZ4 concentration and decreasing the temperature. Figure 3 shows the impact of the temperature on the BZ4 inhibition efficiency. The heat of adsorption for BZ4 adsorption on mild steel was negative, indicating that it is an exothermic process, which explains the decrease in the efficiency with increasing temperature.

### Proposed inhibition mechanism

**Fig. 4:**
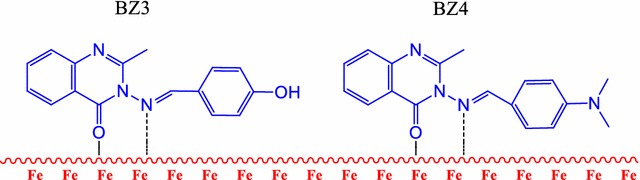
Inhibition mechanism

The efficiencies of the investigated inhibitors BZ3 or BZ4 could rely on charges or molecular weights, in addition to the nature of bonds of the metal and its capability to produce complexes. Figure 4 shows the display complexes formed between the mild steel surface atoms and the investigated inhibitors.

The inhibition mechanism of the tested inhibitors can be explained by valence bond theory (VBT). The Fe^2+^ electron configuration is [Ar]3d^6^. The 3d orbitals mix with the unoccupied 4s and 4p orbitals to form sp^3^ or d^2^sp^3^ hybrid orbitals that might be suitably oriented toward the nitrogen or oxygen non-bonding electron pairs in the inhibitors. When these Fe and inhibitor orbitals overlap, tetrahedral, square planar or octahedral complexes in which the metal has a filled valence shell are formed. The inhibition mechanism can also be explained in terms of crystal field theory (CFT) or molecular orbital theory (MOT). When the inhibitor molecules complex to the metal atoms, coordination bonds form via electron transfer from the inhibitor nitrogen atoms to the metal d orbitals.

### Scanning electron microscopy

**Fig. 5:**
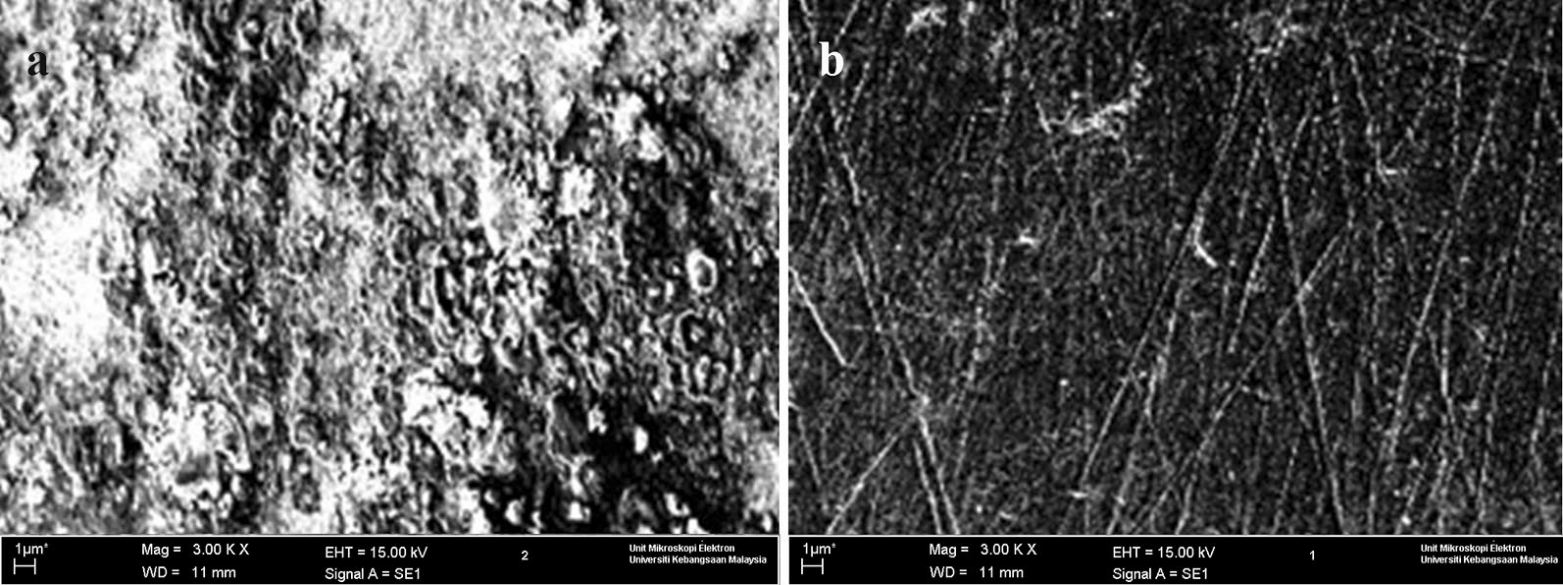
SEM images of mild steel after immersion in a 1.0 M HCl solution **a** without and **b** with BZ4 at 30 °C

The mild steel surface was analyzed by SEM after immersion in 1.0 M HCl with and without 0.5 g/L BZ4 for 3 h at 30 °C, as shown in Fig. 5. After immersion in the HCl solution in the absence of BZ4, the surface appeared to be damaged due to the high iron dissolution rate in corrosive media. However, a barrier was observed on the mild steel surface when BZ4 was added to the solution. This result shows that BZ4 adsorbed on the mild steel surface, protecting it from corrosion by hydrochloric acid.

### DFT studies

To elucidate the significant electronic effects of the substituents, the two inhibitors with strongly electron-donating groups, namely 3-((4-hydroxybenzylidene)amino)-2-methylquinazolin-4(3*H*)-one (BZ3) with a hydroxyl (–OH) group and 3-((4-(dimethylamino)benzylidene)amino)-2-methylquinazolin-4(3*H*)-one (BZ4) with an *N*,*N*-dimethylamino (–NMe_2_) group, were studied by DFT. Two additional isomer models of both BZ3 and BZ4 were also investigated [37].

#### 3-((4-Hydroxybenzylidene)amino)-2-methylquinazolin-4(3*H*)-one (BZ3)

**Fig. 6:**
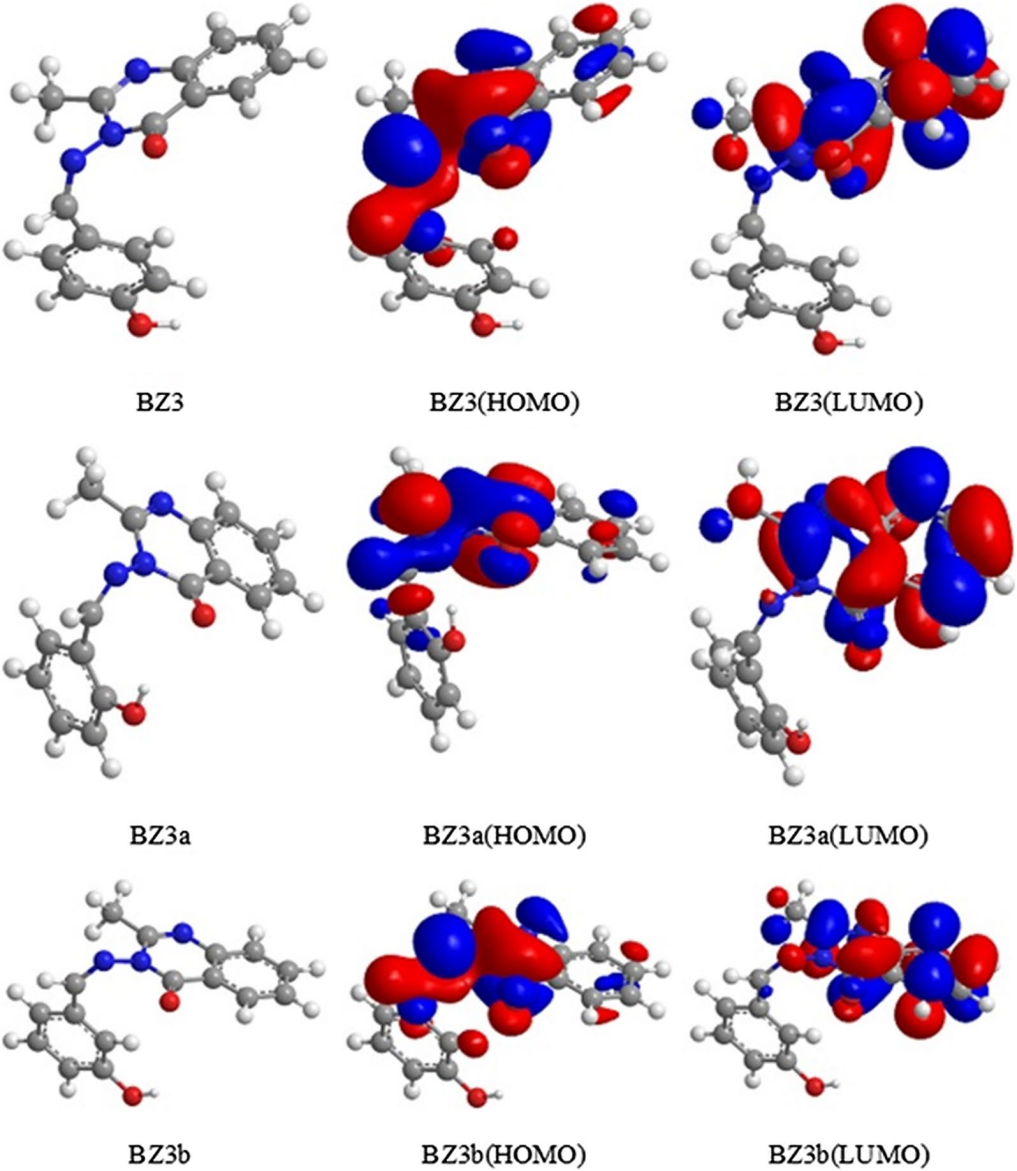
Optimized geometries, HOMOs and LUMOs of BZ3, BZ3a and BZ3b obtained with rB3LYP/6-31G(d,p)

The hydroxyl group on the benzene ring in BZ3 is in the C-4 position but could be moved to the C-2 (BZ3a) and C-3 (BZ3b). For all three positions, the contribution of the substituent to both the HOMO and LUMO was similar with only small variations, as shown in Fig. 6. The optimized geometries of these three isomers are also presented in Fig. 6, and the electronic energies are listed in Table 1.

**Table 1:** Calculated HOMO and LUMO energies, energy gaps, ionization potentials, and electron affinities (eV) for BZ3, BZ3a and BZ3b obtained with rB3LYP/6-31G(d,p)

Molecule	EHOMO	ELUMO	Energy gap (ELUMO − EHOMO)	Ionization potential (I)	Electron affinity (A)
BZ3	− 8.787	− 2.861	5.926	8.787	2.861
BZ3a	− 8.792	− 2.882	5.910	8.792	2.882
BZ3b	− 8.757	− 2.903	5.854	8.757	2.903

The ionization potential (I) and electron affinity (A) were calculated according to Koopmans’ theorem [38, 39] as follows:

I = − EHOMO; A = − ELUMO

The method of Al-Amiery et al. [38, 39] was used to calculate the BZ3 inhibition efficiency (%) from the following equations, and the results are given in Table 2:

2 $$I_{add} \% \; = \;\frac{{I_{BZ3} - I_{X - BZ3} }}{{I_{BZ3} }}\; \times \;100\%$$

3 $$Ie_{add} \% \; = \;I_{add} \% \; \times \;Ie_{BZ3} \%$$

4 $$Ie_{theory} \% \; = \;I_{BZ3} \% \; + \;Ie_{add} \%$$

where *I*_*add*_% is the percent change in the ionization potential of model x-BZ3 relative to that of BZ3, and *Ie*_*add*_% and *Ie*_*theory*_% are the corresponding additional and theoretical inhibition efficiencies, respectively.

**Table 2:** Theoretical inhibition efficiencies (%) for BZ3, BZ3a and BZ3b

Compound	Inhibition efficiency (%)	
	Theoretical (*Ie*_*theory*_)	Experimental
BZ3	92.75	92
BZ3a	96.11	–
BZ3b	77.81	–

These results demonstrate that moving the hydroxyl group to the *meta* position (BZ3b) led to a decrease in the inhibition efficiency to 77.81%, whereas moving it to the *ortho* position (BZ3a) resulted in an increase in the inhibition efficiency to 96.11%. A comparison of the BZ3 and BZ3a inhibition efficiencies (96.11% vs. 92%) reveals that this change in the substituent position clearly enhanced the inhibition efficiency.

#### 3-((4-(Dimethylamino)benzylidene)amino)-2-methylquinazolin-4(3*H*)-one (BZ4)

**Fig. 7:**
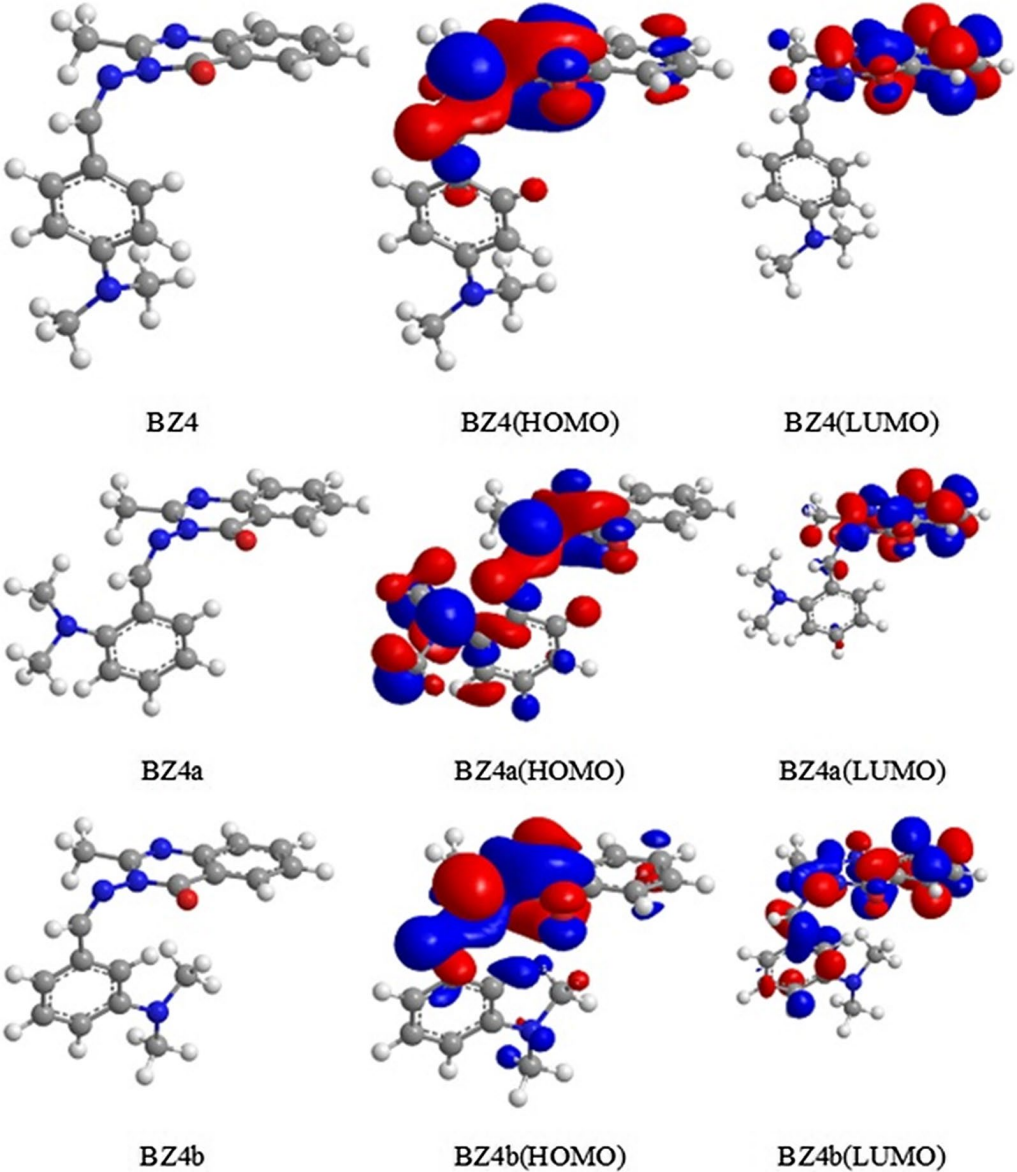
Optimized geometries, HOMOs and LUMOs of BZ4, BZ4a and BZ4b obtained with rB3LYP/6-31G(d,p)

The *N*,*N*-dimethylamine group on the benzene ring in BZ4 is in the C-4 position but could be moved to the C-2 (BZ4a) and C-3 (BZ4b) positions. For all three positions, the contribution of the substituent to both the HOMO and LUMO was similar with only small variations, as shown in Fig. 7. The optimized geometries of these three isomers are also presented in Fig. 7, and the electronic energies are listed in Table 3.

**Table 3:** Calculated HOMO and LUMO energies, energy gaps, ionization potentials, and electron affinities (eV) for BZ4, BZ4a and BZ4b obtained with rB3LYP/6-31G(d,p)

Molecule	EHOMO	ELUMO	Energy gap (ELUMO − EHOMO)	Ionization potential (I)	Electron affinity (A)
BZ4	− 8.787	− 2.855	5.932	8.787	2.855
BZ4a	− 8.843	− 2.855	5.988	8.843	2.855
BZ4b	− 8.646	− 2.919	5.725	8.646	2.919

The ionization potential (I) and electron affinity (A) were calculated according to Koopmans’ theorem [38] as follows:

I = − EHOMO; A = − ELUMO

The inhibition efficiencies of the BZ4 isomers calculated using Eqs. 2–4 are given in Table 4.

**Table 4:** Theoretical inhibition efficiencies (%) for BZ4, BZ4a and BZ4b

Compound	Inhibition efficiency (%)	
	Theoretical (*Ie*_*theory*_)	Experimental
BZ4	95.28	96
BZ4a	94.98	–
BZ4b	85.27	–

These results demonstrate that moving the *N*,*N*-dimethylamino substituent to the *meta* position (BZ4b) led to a decrease in the inhibition efficiency to 85.27%, whereas moving it to the *ortho* position (BZ4a) resulted in an increase in the inhibition efficiency to 94.98%. This result along with that for BZ4 (96%) reveals that an excellent inhibition efficiency could be achieved with BZ4 isomers.

Groups which were withdrawing electron by resonance effect will decrease density of electrons specifically at positions 2, 4 and 6, leaving position 3 and position 5 as the ones with relatively higher efficiency, thus these kinds of groups were (position-3) *meta* directors. Also, the groups that have unoccupied pair of electrons, like the amino group (BZ4) or hydroxyl group (BZ3), are strong active and *ortho* (BZa)/*para*-directors (BZ) thus efficient groups donate the unoccupied electrons to the pi system, making a negative charge on *ortho* (position-2) and *para* (position-4)positions. These positions have the maximum activities toward electron-poor electrophile. The highest electron density have been located on *ortho*/*para* positions, although. An important point; steric hindrance as in compound BZ4 that have 2-methyl groups on nitrogen atom (*N*,*N*-dimethyl) decrease the reactivity. The final result of the electrophilic aromatic substitution might thus be hard to predict, and it is usually only established by doing the reaction and determining the ratio of *ortho* versus *para* substitution.

Finally, from Table 4, BZ4a was less active as inhibitor from BZ due to steric hindrance. From Table 2, the best position was on C-2 (*ortho*-position) for the compound BZ3a and no steric hindrance.

## Conclusions

Mild steel corrosion inhibitors were synthesized, and their structures were fully characterized by spectroscopic techniques. Their abilities to inhibit mild steel corrosion in a 1.0 M HCl solution at 303, 313, 323 and 333 K were subsequently studied. The inhibitors, namely 3-((4-hydroxybenzylidene)amino)-2-methylquinazolin-4(3*H*)-one (BZ3) and 3-((4-(dimethylamino)benzylidene)amino)-2-methylquinazolin-4(3*H*)-one (BZ4), exhibited excellent corrosion inhibition performances, and maximum inhibition efficiencies of 96 and 92% were observed for BZ4 and BZ3, respectively, at an inhibitor concentration of 5 mM. The inhibition efficiency increased with increasing inhibitor concentration, whereas it decreased with increasing temperature. The SEM images show that BZ4 might form a protective film on the mild steel surface.

Quantum chemical calculations were performed to elucidate the relationship between the electronic structures of the inhibitors and their corrosion inhibition efficiencies. In particular, the rB3LYP/6-31G(d,p) calculations of BZ3 and BZ4 isomers revealed that a substituent in the *meta* position on the corrosion inhibitor molecule negatively affected the inhibition efficiency, whereas a substituent in the *para* position enhanced the inhibition efficiency. Compared to other corrosion inhibitors, these molecules exhibited higher inhibition efficiencies. The theoretical and experimental inhibition efficiencies of the studied inhibitors were in excellent agreement, demonstrating the reliability of the method employed.

## References

[1] Ahamad I, Prasad R, Quraishi MA (2010). Thermodynamic, electrochemical and quantum chemical investigation of some Schiff bases as corrosion inhibitors for mild steel in hydrochloric acid solutions. Corros Sci.

[2] Amin MA, Khaled KF, Mohsen Q, Arida HA (2010). A study of the inhibition of iron corrosion in HCl solutions by some amino acids. Corros Sci.

[3] Anitha C, Sheela CD, Tharmaraj P, Shanmugakala R (2013). Studies on synthesis and spectral characterization of some transition metal complexes of azo-azomethine derivative of diaminomaleonitrile. Int J Inorg Chem.

[4] Yıldırım A, Çetin M (2008). Synthesis and evaluation of new long alkyl side chain acetamide, isoxazolidine and isoxazoline derivatives as corrosion inhibitors. Corros Sci.

[5] Emregul KC, Hayvalı M (2004). Studies on the effect of vanillin and protocatechualdehyde on the corrosion of steel in hydrochloric acid. Mater Chem Phys.

[6] Solmaz R (2008). Investigation of adsorption and inhibitive effect of 2-mercaptothiazoline on corrosion of mild steel in hydrochloric acid media. Electrochim Acta.

[7] Bockris JO, Swinkels DAJ (1964). Adsorotion of naphthalene on solid metal electrodes. J Electrochem Soc.

[8] Branzoi V, Branzoi F, Baibarac M (2000). The inhibition of the corrosion of Armco iron in HCl solutions in the presence of surfactants of the type of *N*-alkyl quaternary ammonium salts. Mater Chem Phys.

[9] Oguzie EE, Li Y, Wang FH (2007). Corrosion inhibition and adsorption behavior of methionine on mild steel in sulfuric acid and synergistic effect of iodide ion. J Colloid Interface Sci.

[10] Alijourani J, Raeissi K, Golozar MA (2009). Benzimidazole and its derivatives as corrosion inhibitors for mild steel in 1 M HCl solution. Corros Sci.

[11] Obot IB, Obi-Egbedi NO (2010). Theoretical study of benzimidazole and its derivatives and their potential activity as corrosion inhibitors. Corros Sci.

[12] Li (2006). Synergistic inhibition between o-phenanthroline and chloride ion for steel corrosion in sulphuric acid. Corros Sci.

[13] Kadhum A, Mohamad AB, Hammed L, Al-Amiery AA, San NH (2014). Musa AY Inhibition of mild steel corrosion in hydrochloric acid solution by new coumarin. Materials.

[14] Xia S, Qiu M, Yu L, Liu F, Zhao H (2008). Molecular dynamics and density functional theory study on relationship between structure of imidazoline derivatives and inhibition performance. Corros Sci.

[15] Al-Amiery A, Al-Majedy Y, Kadhum A (2015). Hydrogen peroxide scavenging activity of novel coumarins synthesized using different approaches. PLoS ONE.

[16] Al-Amiery AA, Al-Bayati R, Saour K, Radi M (2012). Cytotoxicity, antioxidant and antimicrobial activities of novel 2-quinolone derivatives derived from coumarins. Res Chem Intermed.

[17] Al-Amiery AA, Al-MajedyKadhum AAH, Mohamad A (2015). Novel macromolecules derived from coumarin: synthesis and antioxidant activity. Sci Rep.

[18] Al-Amiery AA, Al-Majedy YK, Al-Duhaidahawi D, Kadhum AAH, Mohamad AB (2016). Green antioxidants: synthesis and scavenging activity of coumarin-thiadiazoles as potential antioxidants complemented by molecular modeling studies. Free Radic Antioxid.

[19] Al-Amiery AA, Al-Majedy YK, Kadhum AA, Mohamad AB (2014). New coumarin derivative as an eco-friendly inhibitor of corrosion of mild steel in acid medium. Molecules.

[20] Al-Amiery AA, Al-Majedy YK, Kadhum AAH, Mohamad AB (2016). Synthesis of new coumarins complemented by quantum chemical studies. Res Chem Intermed.

[21] Al-Amiery AA, Kadhum AAH, Mohamad AA (2012). Antifungal activities of new coumarins. Molecules.

[22] Al-Amiery AA, Kadhum AAH, Mohamad AB, Musa AY, Li CJ (2013). Electrochemical study on newly synthesized chlorocurcumin as an inhibitor for mild steel corrosion in hydrochloric acid. Materials.

[23] Al-Amiery AA, Musa AY, Kadhum A, Mohamad A (2011). The use of umbelliferone in the synthesis of new heterocyclic compounds. Molecules.

[24] Al-Majedy Y, Kadhum K, Al-Amiery AAH (2014). A synthesis and characterization of some new 4-hydroxy-coumarin derivatives. Molecules.

[25] American Society for Testing and Materials. Standard practice for preparing, cleaning, and evaluating corrosion test specimens. http://www.cosasco.com/documents/ASTM_G1_Standard_Practice.pdf. Accessed 11 Mar 2013

[26] Al-amiery AA, Abdul AHK, Abu BM, Sutiana J (2013). A novel hydrazinecarbothioamide as a potential corrosion inhibitor for mild steel in HCl. Materials.

[27] Junaedi S, Al-amiery A, Kadihum A, Mohamad A (2013). Inhibition effects of a synthesized novel 4-aminoantipyrine derivative on the corrosion of mild steel in hydrochloric acid solution together with quantum chemical studies. Int J Mol Sci.

[28] Ramarajan D, Tamilarasan K, Sudha S (2017). Synthesis, crystal structure analysis and DFT studies of 3a,8a-dihydroxy-2-thioxo-2,3,3a,8a-tetrahydroindeno[1,2-d] imidazol-8(1H)-one. J Mol Struct.

[29] Pandey M, Muthu S, Gowda N (2017). Quantum mechanical and spectroscopic (FT-IR, FT-Raman,1H,13C NMR, UV–Vis) studies, NBO, NLO, HOMO, LUMO and Fukui function analysis of 5-methoxy-1*H*-benzo[d]imidazole-2(3*H*)-thione by DFT studies. J Mol Struct.

[30] Obayes H, Alwan G, Al-Amiery A, Kadhum A, Mohamad A (2013) Thermodynamic and theoretical study of the preparation of new buckyballs from corannulene, coronene, and circulene. J Nanomater 2013;8. Article ID 451920

[31] Sastri VS (1998). Green corrosion inhibitors. Theory and practice.

[32] Olivares-Xometl O, Likhanova NV, Gomez B, Navarrete J, Llanos-Serrano ME, Arce E, Hallen JM (2006). Electrochemical and XPS studies of decylamides of alpha-amino acids adsorption on carbon steel in acidic environment. Appl Surf Sci.

[33] Al-Azawi K, Al-Baghdadi S, Mohamed A, Al-Amiery A, Abed TK, Mohammed SA, Kadhum AA, Mohamad AB (2016). Synthesis, inhibition effects and quantum chemical studies of a novel coumarin derivative on the corrosion of mild steel in a hydrochloric acid solution. Chem Cent J.

[34] Likhanova NV, Martيnez-Palou R, Veloz MA, Matيas DJ, Reyes-Cruz VE, OlivaresXometl O (2007). Microwave-assisted synthesis of 2-(2-pyridyl)azoles. Study of their corrosion inhibiting properties. J Heterocycl Chem.

[35] Chen G, Zhang M, Zhao I, Zhou R, Meng Z, Zhang J (2013). Investigation of ginkgo biloba leave extracts as corrosion and oil field microorganism inhibitors. Chem Cent J.

[36] Fomina L, Porta B, Acosta A, Fomine S (2000). Novel substituted 1-amino-4,5,8-naphthalenetricarboxylic acid-1,8-lactam-4,5-imides: experimental and theoretical study. J Phys Org Chem.

[37] Lukovits I, Kalman E, Zucchi F (2001). Corrosion inhibitors—correlation between electronic structure and efficiency. Corrosion.

[38] Obayes R, Al-Amiery A, Alwan G, Alobaidy A, Al-Amiery A, Kadhum A, Mohamad A (2014). Quantum chemical assessment of benzimidazole derivatives as corrosion Inhibitors. Chem Cent J.

[39] Obayes R, Al-Amiery A, Alwan G, Abdullah T, Kadhum A, Mohamad A (2017). Sulphonamides as corrosion inhibitor: experimental and DFT studies. J Mol Struct.

